# ATF3-Induced Mammary Tumors Exhibit Molecular Features of Human Basal-Like Breast Cancer

**DOI:** 10.3390/ijms22052353

**Published:** 2021-02-26

**Authors:** Leqin Yan, Sally Gaddis, Luis Della Coletta, John Repass, Katherine Leslie Powell, Melissa S. Simper, Yueping Chen, Michelle Byrom, Yi Zhong, Kevin Lin, Bin Liu, Yue Lu, Jianjun Shen, Michael C. MacLeod

**Affiliations:** 1 Department of Epigenetics and Molecular Carcinogenesis, The University of Texas MD Anderson Cancer Center, Science Park, Smithville, TX 78957, USA; lyan@mdanderson.org (L.Y.); salgaddis@mdanderson.org (S.G.); ldcoletta@mdanderson.org (L.D.C.); john@arqgenetics.com (J.R.); powell.leslie@rocketmail.com (K.L.P.); msimper@mdanderson.org (M.S.S.); yuechen@mdanderson.org (Y.C.); mollymolony@gmail.com (M.B.); yizhongjobapp@gmail.com (Y.Z.); kelin@mdanderson.org (K.L.); bliu1@mdanderson.org (B.L.); ylu4@mdanderson.org (Y.L.); 2 Program in Genetics and Epigenetics, MD Anderson Cancer Center UTHealth Graduate School of Biomedical Sciences, The University of Texas MD Anderson Cancer Center, Smithville, TX 78957, USA

**Keywords:** ATF3, mouse model, breast cancer, basal-like, RNA-Seq, miRNA, *Mir145*, *Mir143*

## Abstract

Basal-like breast cancer (BLBC) is an aggressive and deadly subtype of human breast cancer that is highly metastatic, displays stem-cell like features, and has limited treatment options. Therefore, developing and characterizing preclinical mouse models with tumors that resemble BLBC is important for human therapeutic development. *ATF3* is a potent oncogene that is aberrantly expressed in most human breast cancers. In the BK5.ATF3 mouse model, overexpression of *ATF3* in the basal epithelial cells of the mammary gland produces tumors that are characterized by activation of the Wnt/β-catenin signaling pathway. Here, we used RNA-Seq and microRNA (miRNA) microarrays to better define the molecular features of BK5.ATF3-derived mammary tumors. These analyses showed that these tumors share many characteristics of human BLBC including reduced expression of *Rb1*, *Esr1*, and *Pgr* and increased expression of *Erbb2*, *Egfr*, and the genes encoding keratins 5, 6, and 17. An analysis of miRNA expression revealed reduced levels of *Mir145* and *Mir143*, leading to the upregulation of their target genes including both the pluripotency factors *Klf4* and *Sox2* as well as the cancer stem-cell-related gene *Kras*. Finally, we show through knock-down experiments that *ATF3* may directly modulate *MIR145/143* expression. Taken together, our results indicate that the *ATF3* mouse mammary tumor model could provide a powerful model to define the molecular mechanisms leading to BLBC, identify the factors that contribute to its aggressiveness, and, ultimately, discover specific genes and gene networks for therapeutic targeting.

## 1. Introduction

Basal-like breast cancer (BLBC) is one of the most aggressive and deadly subtypes of human breast cancer, primarily affecting mostly young women and marked by highly proliferative cells and high rates of metastasis to the brain and lungs [1]. Notably, 50–80% of human breast cancers express high levels of the bZip transcription factor ATF3 [2,3], and *ATF3* mRNA levels increase when adult cells are exposed to stress signals [4], such as those produced in response to DNA damage [5,6] or other cellular stress [7]. Additionally, immunohistochemical analyses of human breast tumors revealed that ATF3 expression in most tumors is stromal [2] but that 20–25% of human tumors exhibit strong nuclear ATF3 expression in the epithelial compartment of the tumor. These findings suggest that ATF3 may play a role in human breast cancer, particularly in the basal-like subtype of breast cancer.

When overexpressed in the basal epithelial cells of the mouse mammary gland and in the absence of other alterations, *Atf3* acts as an oncogene that can induce frank carcinomas [2]. *Atf3* also promotes metastasis in a xenograft model [3,8] and is required for Ras-mediated transformation in mouse embryo fibroblasts [9]. In the mammary gland, *Atf3*-induced tumorigenesis is accompanied by activation of both the Wnt/β-catenin signaling pathway and the MAPK pathway [10], resulting in the upregulation of Jun and several positive-acting Wnt ligands. Activation of the Wnt/β-catenin signaling pathway has also been associated with mouse tumor initiating cells [11], and MAPK activation has been linked to stem and/or cancer progenitor cell proliferation [12]. These pathways are also commonly activated in BLBC [13,14,15,16,17,18].

Mammary tumors in BK5.ATF3 mice express cytokeratins characteristic of both the luminal (*Krt8*) and myoepithelial (*Krt5*) differentiation pathways [10], suggesting that stem or bipotent progenitor cells may be the target for malignant transformation in this model. Similarly, when a constitutively active β-catenin transgene is overexpressed in basal cells of the mammary gland, it results in the expression of luminal and myoepithelial differentiation pathways [19]. Although the ability of ATF3 to regulate transcription of specific genes has been studied in numerous systems [4,8,20], no previous mechanistic studies have identified Wnt pathway genes or genes that lead to MAPK activation among the targets of ATF3. To molecularly characterize mouse mammary gland tumors and address the possible importance of transcriptional dysregulation in the BK5.ATF3 tumor model, we examined both mRNA and miRNA expression patterns in mammary tumor tissue derived from parous BK5.ATF3 female mice compared to the expression patterns in normal, adjacent mammary glands of the same transgenic mice. We compared these expression patterns to the gene expression patterns of five expression-defined subtypes of human breast cancers [21], and to the expression patterns of 27 genetically engineered mouse models of human breast cancer [22]. We present several lines of evidence supporting the idea that the dysregulated expression patterns of individual genes and enriched molecular pathways in these BK5.ATF3 mammary gland tumors are consistent with the hallmarks of basal-like human breast cancer. We have also identified several dysregulated miRNAs that appear to be involved in the expansion of stem or progenitor cell populations in murine mammary glands [23]. Taken together, our results indicate that the BK5.ATF3 mouse model may provide a robust model for investigating the cellular mechanisms that contribute to BLBC.

## 2. Results

### 2.1. Gene Expression Pattern of ATF3-Induced Mammary Tumors is Consistent with Characteristics of Human Basal-Like Breast Cancers

**Figure 1:**
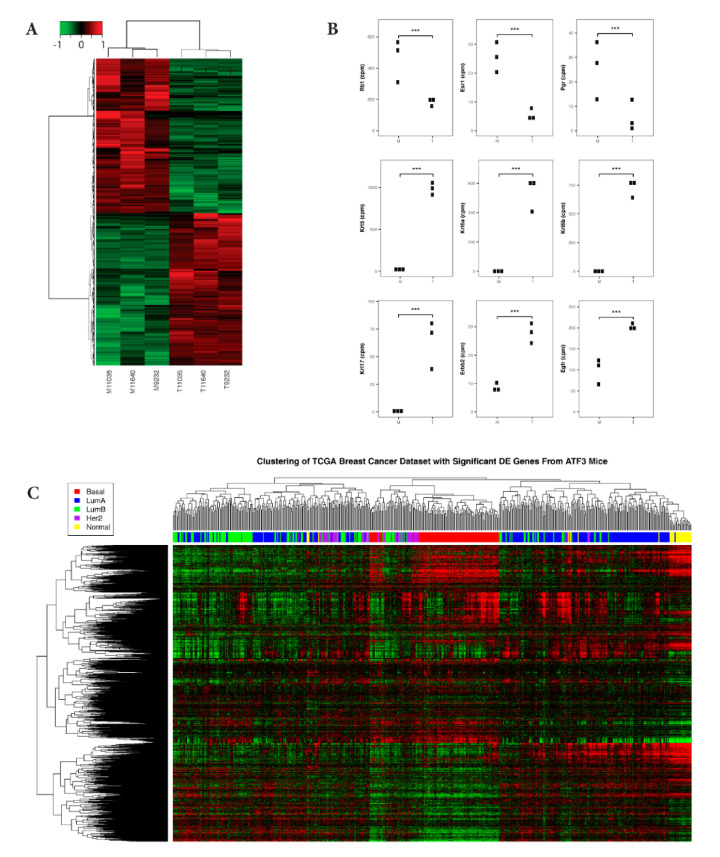
Gene expression patterns of ATF3-induced tumors are consistent with those of human basal-like breast cancer (BLBC). (**A**) Unsupervised hierarchical clustering and heatmap presentation of all DEGs were generated from three pairs of matched, adjacent, normal mammary glands (M) and mammary tumors (T) from BK5.ATF3 transgenic mice using the hclust and heatmap2 functions in R with gene expression values calculated using edgeR. Red represents upregulated genes and green represents downregulated genes. The expression values are log2 fold changes between tumor and adjacent normal tissues (fold change ≥ 2.0, FDR ≤ 0.05). (**B**) Dot plots showing the difference in expression of selected genes in BK5.ATF3 mice for both normal tissue (M) and tumor tissue (T). *** indicates a significance of FDR < 0.005. (**C**) Unsupervised hierarchical clustering and heatmap presentation comparing expression of 3979 differentially expressed genes (DEGs) identified in the BK5.ATF3 mammary tumors and their human homologs. The expression profiles for the 3979 genes in 547 human breast cancer samples representing Basal-like, LuminalA, Luminal B, Her2, and Normal-like subtypes were compared with the mouse DEGs. The colored bar across the top indicates the subtypes of the patient samples depicted in the figure key: Basal-like (red), LumA (blue), LumB (green), Her2 (purple), and Normal (yellow). The horizontal dendogram on the top represents the 547 patient samples, and the vertical dendogram on the left represents 3979 DEGs.

We performed RNA-Seq analysis on single mammary tumors from each of three parous, transgenic BK5.ATF3 mice, along with matched normal, adjacent, mammary gland tissue from the same transgenic mice. We identified 4739 differentially expressed genes (DEGs) (with FC ≥ 2 and FDR ≤ 0.05) using edgeR. These genes were subjected to an unsupervised hierarchical clustering analysis, which showed a clear separation of DEGs in mammary gland tumors compared to the adjacent control tissues (Figure 1A). Previously, we identified a number of Wnt/β-catenin signaling pathway genes and their targets in this BK5.ATF3 tumor model that are differentially expressed [10]. The changes in expression patterns of nine of these previously identified genes (*Wnt3*, *Wnt5a*, *Wnt5b*, *Wnt7b*, *Wnt10b*, *Ctnnb1, Ccnd1, Jun*, and *Axin2*) were validated in this new RNA-Seq data set (Supplementary Figure S1). In addition, *Snai1*, implicated in the epithelial-mesenchymal transition (EMT), and *Snai2* were both significantly up-regulated (Supplementary Figure S1). Importantly, and consistent with our data, both Wnt/β-catenin signaling and EMT are known to be up-regulated in human BLBC [21]. In addition, we observed changes in the expression of specific genes, including significant reductions in the expression of *Rb1*, *Esr1*, and *Pgr*, and increases in the expression of *Erbb2*, *Egfr*, and the genes encoding keratins 5, 6, and 17 (Figure 1B), as they are in human basal-like breast tumors (Cancer Genome Atlas 2012).

In order to better characterize the BK5.ATF3 mammary tumors, we compared the DEGs we identified in the ATF3 mouse tumors to human breast cancer gene expression profiles [21]. Among the 4739 differentially expressed mouse tumor genes, 3979 human homologs were identified. Expression data for these homologs were retrieved for 547 human samples from the human breast cancer database (https://gdc.cancer.gov/node/877 (accessed on 1 December 2020)) [21]. These human samples were clustered into five PAM50 subtype groups (Basal-like, Her2, LumA, LumB, and Normal) [21]. Unsupervised hierarchical clustering of the human and mouse expression data illustrated that human samples from the basal-like subtype group clustered with the ATF3 tumors, whereas other human breast cancer subtypes did not (Figure 1C). This indicates that the BK5.ATF3 mouse tumors share gene expression characteristics with the human basal-like subtype group (Supplementary Figure S2).

### 2.2. EMT, Wnt Signaling, and Stem Cell Biology are Implicated in ATF3-Induced Mammary Tumors

**Figure 2:**
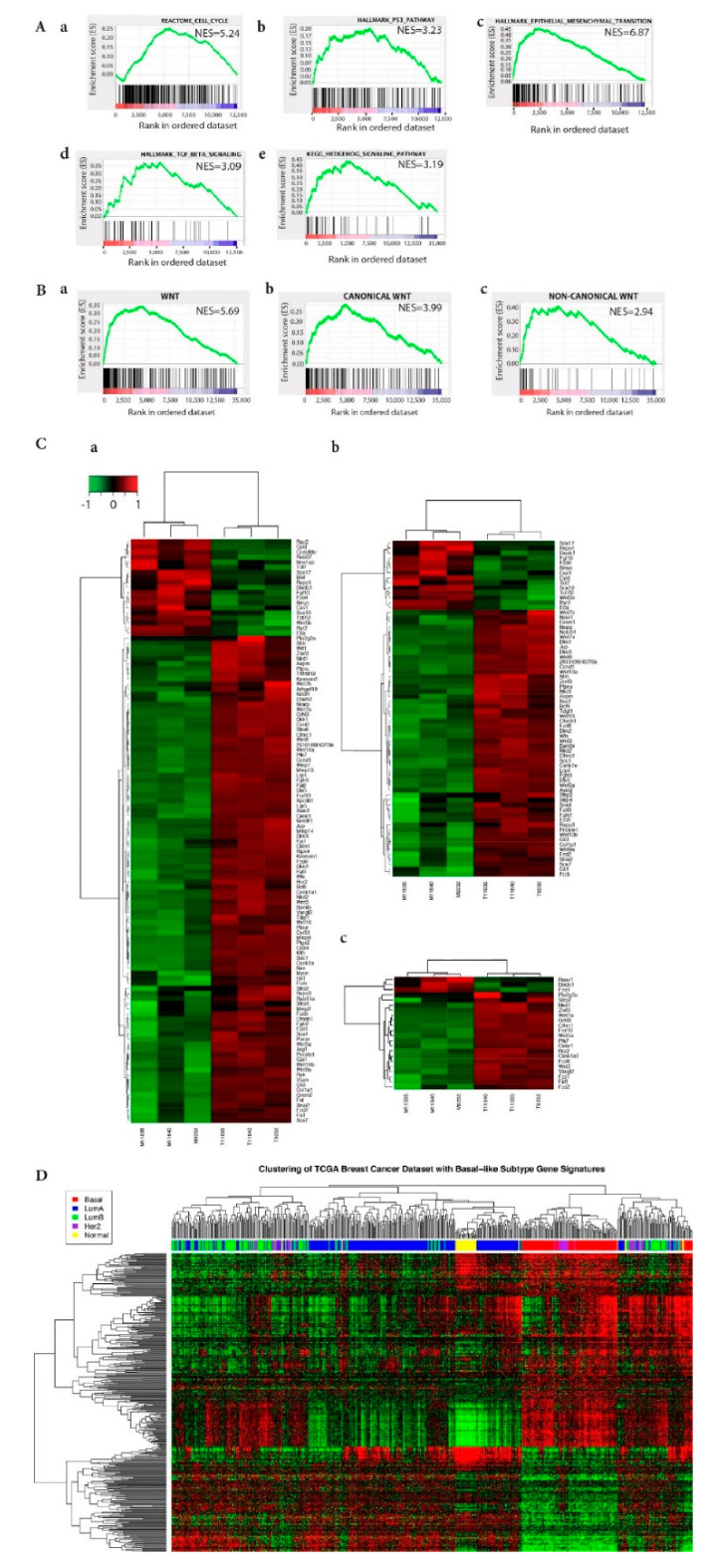
Signaling pathway signatures of ATF3-induced tumors are similar to those of human BLBC. (**A**) GSEA enriched gene sets cell cycle (a), p53 (b), EMT (c), TGF-beta (d), and Hedgehog signaling pathway (e). (**B**) GSEA enriched gene sets Wnt pathways (a), canonical Wnt pathway (b), and non-canonical Wnt pathway (c). (**C**) Heatmaps showing the expression of all Wnt pathway genes in BK5.ATF3 mice (a), expression of canonical Wnt genes in mouse tumors (T11035, T11640, T9232) and matched non-tumor, transgenic mammary gland tissue (M11035, M11640, M9232) (b), and expression of non-canonical Wnt genes, as in b (c). (**D**) Heatmap showing the expression of genes in common between the BK5.ATF3 mouse model and the GSEA-identified enriched genes from human basal-like breast cancer (BLBC) sample data contained in TCGA using the human biological process gene sets in Gene Ontology. For (**A**) and (**B**), the GSEA analysis was performed using GSEAPreranked (Subramanian, Tamayo et al. 2005) against gene sets from V5.1 of the Molecular Signatures Database (MSigDB). Gene pre-ranking was based on the *p*-value obtained from the edgeR analysis. For (**A**), gene sets are from V5.1 of the Molecular Signatures Database (MSigDB). For (**B**), the Wnt pathway gene set (a) was compiled based on GO Ontology, DAVID, the Wnt Homepage, and the scientific literature. The Wnt pathway gene set (a) was sub-categorized into canonical Wnt (b) and non-canonical Wnt (c) gene sets.

To identify the pathways and networks significantly enriched within the DEGs, we performed Ingenuity Pathway Analysis (IPA) and Gene Set Enrichment Analysis (GSEA) on the expression data. The GSEA study incorporated the KEGG, Hallmark, Biocarta, and Reactome gene sets from the Molecular Signatures Database and revealed a strong enrichment for the cell cycle and cell cycle checkpoint-related genes in the KEGG, Biocarta, and Reactome gene sets (Figure 2Aa shows the Reactome Cell Cycle analysis). This is consistent with findings for basal-like human breast cancers with high proliferation rates [21]. In addition, the p53 signaling pathway, central to many cellular processes that respond to DNA damage and cell stress, was significantly enriched in the Hallmark, KEGG, and Reactome gene sets (Figure 2Ab shows the Hallmark p53 pathway).

Genes involved in the epithelial-mesenchymal transition (EMT) were the top Hallmark-enriched gene set (Figure 2Ac) and many genes required for EMT are upregulated in BLBC [24,25]. Furthermore, EMT may require both endogenous, cell autonomous signaling (TGF-β, Hedgehog, Notch, and Wnt) and exogenous, non-cell autonomous signaling [26]. Consistent with this idea, the TGF-β, Hedgehog, and Wnt pathways were significantly enriched (Figure 2Ad,Ae,Ba), as was the Notch pathway, albeit to a lesser extent (data not shown). Importantly, Hedgehog, Wnt, and Notch signaling pathways are dysregulated in breast cancer and thought to play an important role in the regulation and maintenance of stem cells [27]. Individually, TGF-β plays a role in the metastasis of BLBC to bone and lung [28], and hedgehog signaling is activated in human mammary stem cells by EMT programs [29].

The canonical Wnt/β-catenin signaling pathway is upregulated and/or activated in ATF3-induced mammary tumors [10], which may affect tumor heterogeneity in BLBCs [30,31] and GSEA revealed that, in addition to enrichment of the canonical Wnt pathway, non-canonical Wnt pathways were also enriched in ATF3-induced mammary tumors (Figure 2Bb,Bc). We compiled a list of 240 unique mouse genes implicated in either the canonical or non-canonical Wnt signaling pathways based on GO Ontology, DAVID, the Wnt Homepage, and the scientific literature. Of these 240 genes, 115 genes (47.9%) were significantly differentially expressed (*p* = 8 × 10^−22^8e-22) in a tumor compared to adjacent control mammary gland tissues (Figure 2C), with 96 genes (83.5%) being upregulated (Figure 2Ba,Bc,Ca,Cc). In contrast to this sharp upregulation of Wnt signaling genes, in the overall dataset, DEGs were split evenly between upregulated (2349) and downregulated (2390) genes (Figure 1A). IPA confirmed that the DEGs were involved in pathways linked to Wnt/β-catenin signaling (Supplementary Figure S3). Notably, genes involved in human embryonic stem cell pluripotency were also among the top upregulated pathways revealed by this analysis.

In order to narrow the list of signaling pathways, we used GSEA to identify gene sets that are both enriched in BLBC, based on the GO biological process databases, and differentially expressed in BK5.ATF3 mice. A total of 1003 genes enriched in BLBC (FDR < 0.05) was then intersected with the 3979 differentially expressed mouse genes for which we had identified human homologs (Figure 1C). This led to the recovery of 405 genes that were both enriched in BLBC and differentially expressed in BK5.ATF3 mice. Next, we performed unsupervised hierarchical clustering of 547 human breast cancer samples with these 405 genes (Figure 2D) and found co-clustering of the BLBC samples and the differentially expressed mouse tumor genes. This result indicates that sets of genes thought to be important in the biology of human BLBC are differentially regulated in BK5-ATF3-induced tumors in much the same way, and confirms the similarity between this murine model and human BLBC.

### 2.3. ATF3 Mammary Tumors Resemble Three Basal-Like Breast Cancer Mouse Models

**Figure 3:**
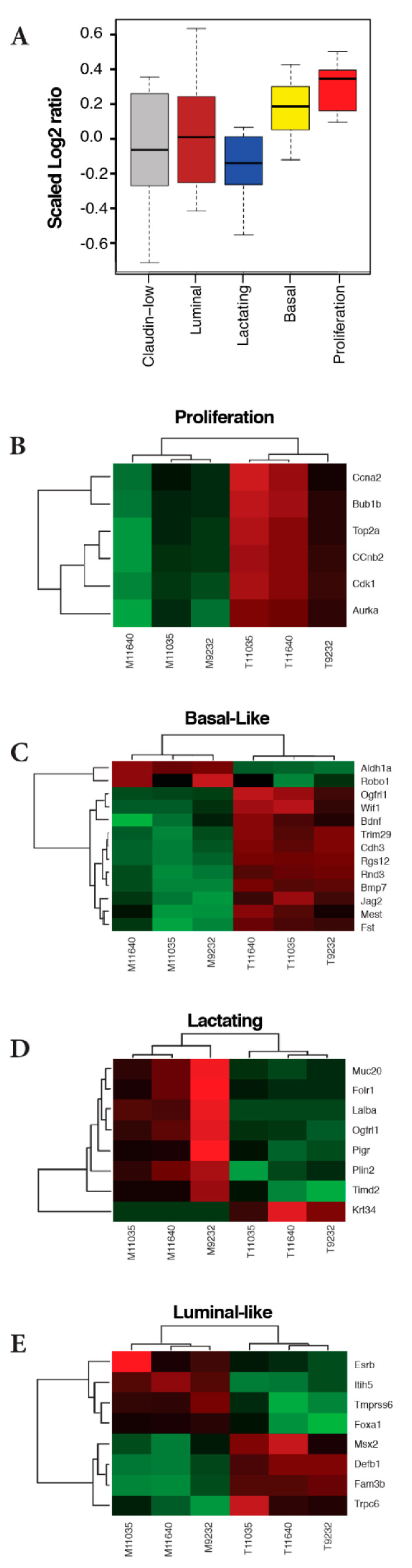
Comparison of BK5-ATF3 tumor data based on the expression of five intrinsic gene sets used to characterize murine models of breast cancer [22]. (**A**) Boxplot of expression log2ratio values for each mouse gene subtype signature (proliferation, basal-like, lactating, luminal, and claudin-low). The log2ratio values were from RNA-Seq and scaled so that the sum of squares in each subtype is 1.0. Only the proliferation and basal gene sets had average log2 ratios significantly different than zero (*p* < 0.05). (**B**–**E**) Heatmaps depicting four of the five gene sets. (**B**) Proliferation. (**C**) Basal-like. (**D**) Lactating. (**E**) Luminal-like. For the boxplots, all the genes of each subtype were used. For the heatmap, the significantly differential genes of each subtype in RNA-Seq were used.

In an extensive analysis of genetically engineered mouse models of human breast cancer, Pflefferle and colleagues used selected cell lineage markers to identify intrinsic gene expression sets that characterized five classes of murine models: claudin-low, luminal, basal, proliferation, and lactating [22]. Human BLBC tumors tend to overexpress genes in two of these intrinsic murine expression sets: basal and proliferation. We calculated the normalized expression values (tumor: normal) in our ATF3-induced tumors for each of these intrinsic gene expression sets, and found that only the basal and proliferation gene sets were significantly overexpressed (*p* = 0.0081 and 0.00019, respectively) (Figure 3A). Heat maps for four of these gene sets are shown (Figure 3, panels B–E). Together, these results indicate that the gene expression pattern of the BK5.ATF3 mouse model tumors is similar to the expression patterns of three other basal-like murine breast cancer models: C3Tag^Ex^, Myc^Ex^, and p53null-Basal^Ex^ [22] and consistent with the signature of highly proliferative, human basal-like tumors [21].

### 2.4. Identification of Differentially Expressed miRNAs

**Figure 4:**
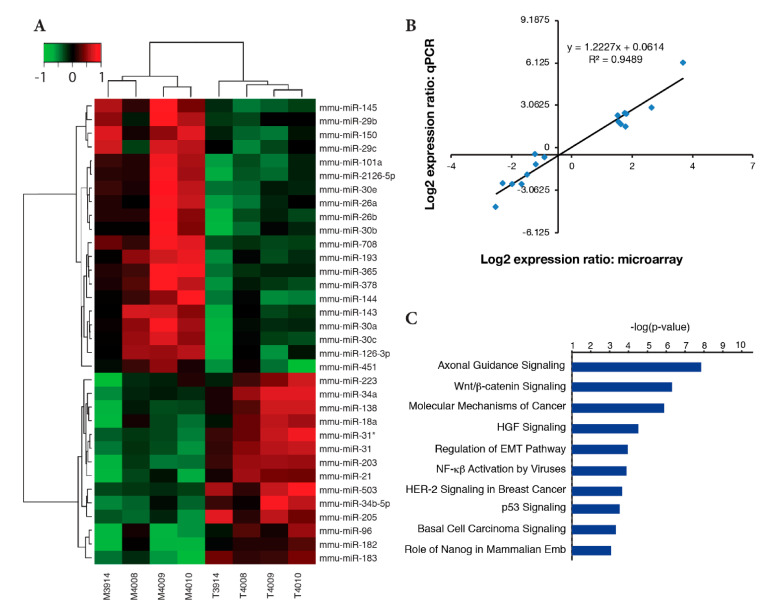
Identification and validation of differentially expressed miRNA genes. (**A**) Heatmap of 34 significantly differentially expressed miRNA genes between four pairs of matched tumors (T) and their adjacent mammary tissues (M) from ATF3-expressing mammary glands. (**B**) Correlation of gene expression between miRNA genes identified by a microarray and selected for validation with RT-qPCR assays using RNA from a total of six tumors and six paired, normal, mammary gland controls, including the four pairs used for the microarray experiments. (**C**) IPA analysis of the integrated mRNA and miRNA datasets.

We compared the miRNA profiles from a set of four independent BK5.ATF3 mammary tumors to those of paired normal, transgenic mammary tissues using miRNA microarrays. To focus the list of differentially-expressed miRNAs on those most characteristic of the mammary tumors, we considered only those that were upregulated or downregulated ≥ 2-fold in at least three of the four tumors. Based on these criteria, we identified 34 differentially expressed miRNA genes, including *Mir145* and *Mir143* (Supplementary Table S1). Of these 34 genes, 14 were significantly upregulated and 20 were significantly downregulated. Relative expression levels for each of these genes, in each of the four tumor/normal pairs, were in agreement between tumors for this gene set (Figure 4A). A subset of 16 differentially expressed miRNAs was validated by RT-qPCR using six tumor samples and their paired normal, transgenic mammary gland samples, including those examined by a microarray. The correlation between the microarray results and the qPCR results was excellent with a Pearson correlation coefficient of 0.949 (Figure 4B).

Many miRNAs have been implicated in the initiation or progression of cancer, but defining the biological processes these miRNAs regulate has been more difficult. We took an integrated approach using IPA to simultaneously assess differentially expressed pairs of miRNAs and mRNAs based on: 1) whether the targeting of a specific miRNA on an mRNA had been experimentally validated or predicted with high confidence, and 2) whether the change in expression of the miRNA and mRNA were in opposite directions. We found that genes involved in Wnt/β-catenin signaling, EMT regulation, p53 signaling, and Nanog-related pathways involved in embryonic stem cell pluripotency were enriched in mammary tumors vs. normal transgenic mammary tissue (Figure 4C). Overall, seven networks were identified from this integrated analysis. Supplementary Figure S4 shows one of these networks, in which three miRNA modules were identified, including one centered on *Mir145* (the other networks are not shown). We chose *Mir145* and *Mir143*, which are transcribed as a single precursor molecule and are involved in regulating stem cell pluripotency [32] for further analysis.

### 2.5. Reduced Mir145 and Mir143 Expression in ATF3 Tumors Upregulates Transcription Factors Klf4 and Sox2 and Cancer Stem Cell-Related Gene Kras

*Mir145* expression was downregulated ~6-fold in ATF3-induced mammary tumors (Figure 4A and Supplementary Table S1), consistent with its downregulation in human breast cancers [33,34,35], including basal-like [36] and triple-negative [37] breast cancers.

**Figure 5:**
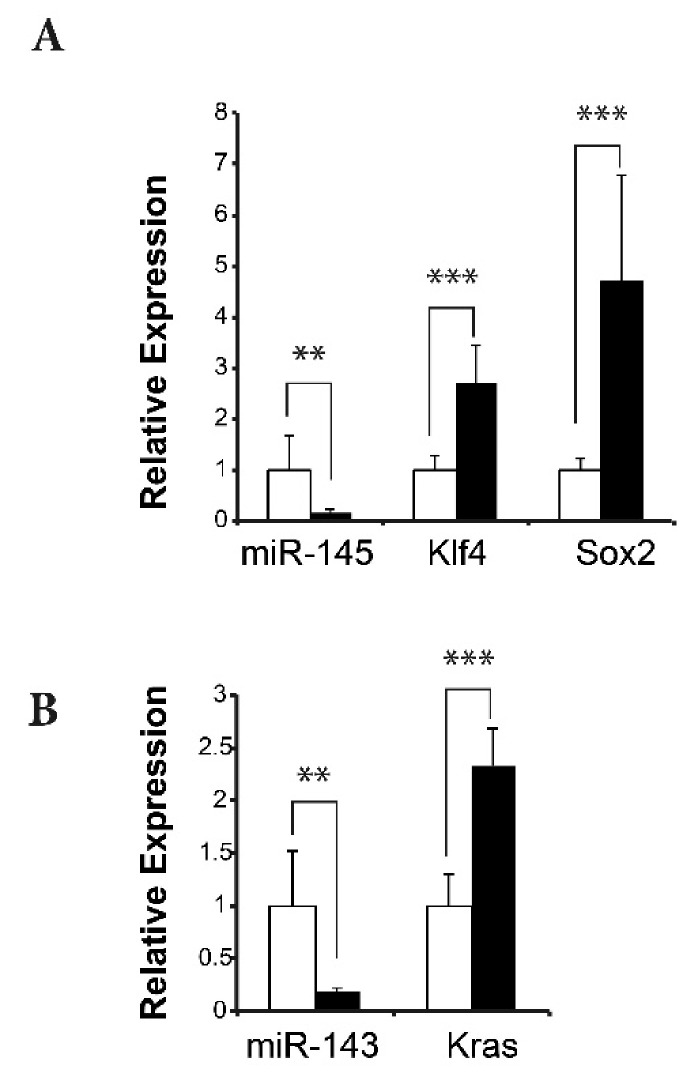
Expression of miR-145 and miR-143 and their downstream, stem cell-related gene targets in normal, transgenic mammary tissues (white) and ATF3-induced tumors (black). (**A**) Relative expression of miR-145 and the pluripotency factors *Klf4* and *Sox2*. (**B**) Relative expression of miR-143 and its target gene *Kras*. ** *p* < 0.05. *** *p* < 0.005.

In humans, the transcription factors KLF4, SOX2, OCT4, and MYC are important for maintaining the self-renewal capacity of embryonic stem cells and contain 3′UTR sequences that are directly targeted by MIR145 to regulate expression [38,39]. These sites are conserved in the murine *Klf4* and *Sox2* mRNAs, but not in the *Oct4* and *Myc* mRNAs. We tested whether expression of either *Klf4* or *Sox2* was affected in BK5.ATF3 mammary tumors using RT-qPCR and found that both *Klf4* and *Sox2* were significantly upregulated (*p* < 0.005) in tumors with lower *MiR145* levels (Figure 5A).

Notably, *Mir143* was downregulated nearly five-fold in ATF3-induced tumors (Figure 4A and Supplementary Table S1), likely as a consequence of *Mir143* and *Mir145* being transcribed as a single precursor molecule [32]. *Kras* is both a target of *Mir143* [40] and cancer stem cell-related. Therefore, we looked for expression changes in *Kras* in ATF3-induced tumors and found a ≥two-fold increase (*p* < 0.0001) in expression (Figure 5B).

### 2.6. Effects of ATF3 Knockdown on the Expression of MIR145/143

**Figure 6:**
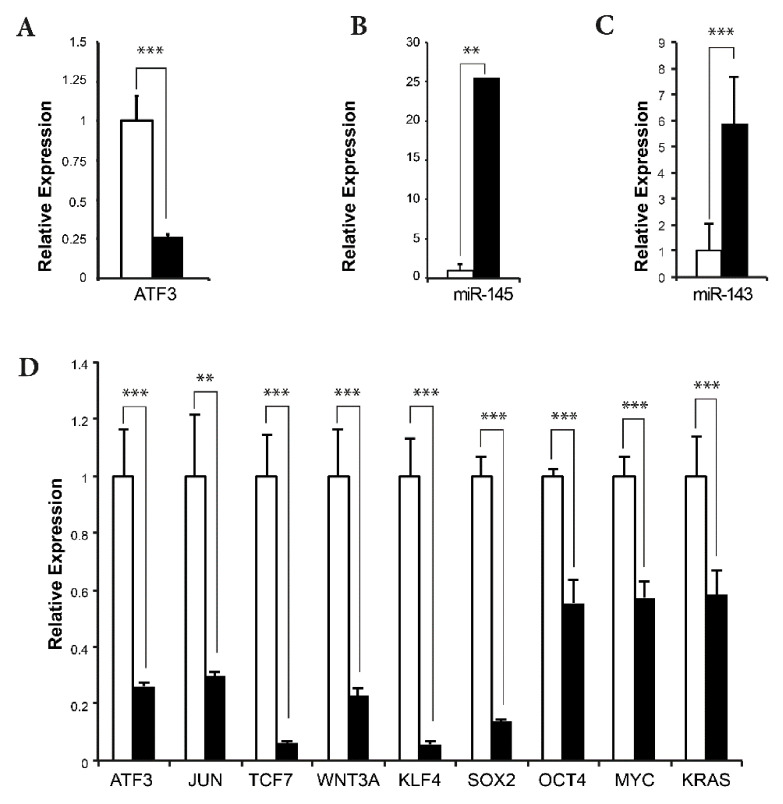
Effects of ATF3 knockdown on the expression of selected genes in the TNBC cell line MDA-MB-157 determined by RT-qPCR. (**A**) Plot showing the relative expression of *ATF3* in the presence of either a scrambled shRNA (white), or an ATF3 shRNA (black). (**B**) As in A, but for miR-145. (**C**) As in A, but for miR-143. (**D**) As in A but for *ATF3*, *JUN*, *TCF7*, *WNT3A*, *KLF4*, *SOX2*, *OCT4*, *MYC*, and *KRAS*. ** *p* < 0.05, *** *p* < 0.005.

To determine whether ATF3 directly affects expression of *MIR145/14*3 in human breast cancer cells, we generated an shRNA knockdown system to experimentally manipulate *ATF3* levels in MDA-MB-157 cells. MDA-MB-157 is a triple-negative human breast cancer cell line that expresses high levels of ATF3. The phenotypes associated with TNBC include MAPK activation, activation of Wnt/β-catenin signaling, and a basal-like cytokeratin expression pattern, similar to the phenotypes of BK5.ATF3 mammary tumors. Knockdown of ATF3, confirmed by immunoblotting (data not shown), resulted in a 60–70% reduction of *ATF3* mRNA levels (Figure 6A) and a ~25-fold increase in the expression of both *MIR145* (Figure 6B) and *MIR143* (Figure 6C), relative to controls, as measured by RT-qPCR. Expression of four downstream targets of *MIR145* was also significantly decreased (*p* < 0.001) following ATF3 knockdown, ranging from an 85–90% decrease for *KLF4* and *SOX2* to a 50% decrease for *MYC* and *OCT4* (Figure 6D).

## 3. Discussion

Basal-like breast cancer is one of the most aggressive and deadly human breast cancers and is marked by a high incidence of recurrence and a high frequency of metastasis [41]. Here, we have shown that ATF3-induced mammary tumors in BK5.ATF3 mice have gene expression characteristics similar to both human BLBC and other “basal-like” mouse models of mammary cancer.

At the individual gene level and compared to controls, ATF3-induced mammary tumors exhibited reduced expression of *Rb1*, *Esr1*, and *Pgr*, and increased expression of *Erbb2*, *Egfr*, and the genes encoding keratins 5, 6, and 17 (Figure 1B), which is similar to basal-like human breast cancer tumors [42,43]. At the pathway and/or network level, GSEA and IPA analyses showed that signaling pathways characteristic of BLBC were enriched in the BK5.ATF3 mouse model. Within the Hallmark gene set, EMT and P53 pathway genes were strongly enriched, as were the TGF-β, Wnt/β-catenin, TNFα (via NFkB), and Notch signaling pathways, indicating that EMT and its related signaling pathways were activated in these ATF3 mammary tumors. Cell cycle genes and pathways were enriched in both the Biocarta and Reactome gene sets (Figure 1 and Figure 2) as they are in human BLBC [26]. Importantly, some of the significantly enhanced pathways/networks in these mammary tumors were similar to those of three basal-like murine breast cancer models, C3Tag^Ex^, Myc^Ex^, and p53null-Basal^Ex^ (Figure 3A–E) [22], as well as the gene expression profiles of human breast cancer patients (Figure 1C and Figure 2D) [21]. Together, our results suggest that the BK5.ATF3 transgenic model strongly reflects human basal-like breast tumors.

Although the BK5.ATF3 model shares many molecular features with the C3Tag^Ex^, Myc^Ex^, and p53null-Basal^Ex^ mouse models, it also has several other uniquely enriched gene sets that are characteristic of human BLBC, including genes involved in EMT, TGF-β signaling, Wnt/β- catenin signaling, Hedgehog signaling, and Notch signaling. These ATF3-specific enriched gene sets and other factors, such as differentially expressed miRNA genes, distinguish this model from the other mouse BLBC models, making it a complementary model for dissecting the molecular mechanisms impacting human basal-like breast tumors, particularly with regard to chemoresistance and the capacity to migrate, invade, and metastasize [as reviewed by [26]]. In this regard, two of the 34 differentially expressed miRNA genes we identified, *Mir145* and *Mir143*, appear to be involved in the expansion of stem or progenitor cell populations in the mammary gland [23].

Two earlier studies demonstrated that *MIR145* directly regulates a group of transcription factors that includes KLF4, SOX2, OCT4, and MYC, which are important for maintaining the self-renewal capacity of embryonic stem cells [38,39]. We found that *Mir145* was significantly downregulated in ATF3-induced mammary tumors, and that its direct targets *Klf4* and *Sox2* were significantly upregulated (Figure 5A). Similarly, *Mir143* was downregulated, and its target *Kras* was upregulated (Figure 5B). Furthermore, we discovered that two other cancer stem cell-related genes, *Cd44* and *Itga6*, were also significantly upregulated in the tumors (Supplementary Figure S5). To show that ATF3 was directly involved, we knocked down the expression of ATF3 in human MDA-MB-157 cells and demonstrated that reduced ATF3 expression upregulated *MIR145* and *MIR143* expression, which led to downregulation of *KLF4*, *SOX2*, *OCT4*, *MYC*, and *KRAS*. Taken together, these results are consistent with a model in which ATF3 overexpression in the ATF3 mouse mammary tumors suppresses *Mir145* and *Mir143* expression, which, in turn, activates mammary cancer stem cells or stem cell-like cells to self-renew and turns on the expression of pluripotency genes [39]. Preliminary FACS analysis utilizing stem cell markers CD24 and CD49f, indicates a substantial expansion of the stem-like cell compartment in transgenic mammary glands (unpublished data, M.C.M. and K.L.P.).

In addition to identifying *Mir145* and *Mir143*, we confirmed the previously reported enrichment/upregulation of both canonical and non-canonical Wnt/β-catenin signaling pathways in the ATF3 mouse model [10]. The activation and/or dysregulation of the Wnt signaling pathway, along with alterations in TGF-β, Hedgehog, and Notch signaling have also been reported for human BLBC [26,27]. Together, these pathways constitute the main endogenous pathways of EMT in BLBC cells [26]. These signaling pathways also play an important role in regulation and maintenance of stem cells [27], suggesting that they may be important in the maintenance and/or regulation of stem-like breast cancer cells. Consistent with this notion, DiMeo and colleagues showed that the inhibition of Wnt signaling suppresses the ability of BLBC cells to self-renew [15]. Taken together, our results suggest that ATF3 activates the Wnt signaling pathway and other signaling pathways involved in the EMT transition and, therefore, may induce EMT itself. That is, Wnt signaling enables stem cell self-renewal through the EMT transition in ATF3-induced mammary tumors.

## 4. Materials and Methods

### 4.1. Animals

Mice were maintained in an AAALAC-accredited facility with temperature and light control, and mice were provided access to water and lab chow ad libitum. Experimental procedures were approved by the Institutional Animal Care and Use Committee of the University of Texas MD Anderson Cancer Center under protocol #05–01-03934. The derivation of the BK5.ATF3 transgenic mouse strain has been described [44]. Mammary tumors arose in parous BK5.ATF3 transgenic females between 6 and 12 months of age, and animals were sacrificed when their tumors reached 1.5 cm in their longest dimension.

### 4.2. Cell Culture

The human breast cancer cell line MDA-MB-157 (ATCC:HTB-24) was maintained in Leibovitz L-15 medium containing 10% FBS at 37 °C in 5% CO_2_. MDA-MB-157 cells were transfected with either shRNA vectors designed to knock-down ATF3 expression in human cells (157KD1) or a scrambled shRNA control (157SCR) vector.

### 4.3. miRNA Microarray and RT-qPCR Gene Expression Analysis

Total RNA was prepared from mammary tumors and from matched, adjacent, normal transgenic mammary glands. Exiqon microarrays (Qiagen Inc, Germantown, MD) were used to assess differential miRNA expression. Four independent sets of tumors and their matched, normal transgenic mammary glands were compared. Three sets were assayed in duplicate (dye swap) and one set was tested in only a single assay. The dye swap assay was not performed for this pair. Total RNA (1000 ng) was labeled using either Hy5 or Hy3 dye following the manufacturer’s protocol for the miRCURY LNA microRNA Array (Exiqon/Qiagen). The Hy5-labeled samples and Hy3-labeled samples were mixed pairwise and hybridized to the array. Hybridization and wash steps were performed using a Tecan HS400 Hybridization Station (Tecan, Austria). The miRCURY LNA array slides were scanned using GenePix 4200 (Molecular Devices, San Jose, CA). Differential miRNA expression was validated by reverse-transcriptase quantitative PCR (RT-qPCR). RT-qPCR was performed utilizing SYBR assays obtained from Exiqon (Qiagen Inc.), using the same four pairs of tumors and matched tissues that were used for the microarray experiments. These experiments also included two additional tumor-matched tissue pairs. Expression of potential downstream target genes was assessed using RT-qPCR.

### 4.4. mRNA RNA-Seq Sequencing

For RNA-Seq analysis, three independent sets of mammary tumors and matched adjacent, normal, transgenic mammary glands, which were different from the six pairs used in the microarray and/or RT-qPCR validation experiments, were used to isolate total RNA. One hundred nanograms of total RNA from each sample were used to prepare libraries using the Ovation RNA-Seq System V2 (NuGEN Technologies, Inc., San Carlos, CA). The six libraries were sequenced in one lane (2 × 76 bp paired-end run) on an Illumina HiSeq 2500 instrument.

### 4.5. Bioinformatics Analysis

#### 4.5.1. MiRNA Microarray Analysis

MicroRNA differential expression analysis was conducted in R V3.2.3 [45] using the limma package V3.26.8 [46]. Low quality and control spots were filtered and the background was corrected by subtraction. Inter-array normalization was performed using variance stabilizing normalization [47]. Within-array duplicate spot correlation was determined using limma duplicate correlation [48]. Fold change estimates and standard errors were calculated using limma lmFit [49]. Empirical Bayes smoothing was applied to the standard error values, and *p*-values were adjusted with the Benjamini & Hochberg method [50]. Genes that were differentially expressed in the four tumors (*p* value < 5 × 10^−5^, B-statistic > 4.6) relative to their paired normal tissues were selected and ranked by a fold change. MiRNA genes that showed at least a two-fold expression difference in at least three of the four tumors were selected for further study.

#### 4.5.2. RNA-Seq Data Analysis

For the RNA-Seq experiment, 25–32 million pairs of reads were generated per sample with each pair of reads representing a cDNA fragment from the library. The reads were mapped to the mouse genome (mm9) using TopHat [51] (V2.0.3). The overall fragment mapping rates ranged from 84–95%. The number of fragments corresponding to each known gene from the RefSeq database [52] (downloaded from UCSC Genome Browser on 9 March 2012) was enumerated using htseq-count from the HTSeq package [53] (V0.5.3p9). Genes represented by fewer than 10 fragments across all samples were removed before differential expression analysis. Differential gene expression was statistically assessed using the R/Bioconductor package edgeR (V3.6.1) [54]. Genes with FDR (false discovery rate) ≤ 0.05, FC (fold change) ≥ 2, and length > 200 bp were called differentially expressed. Hierarchical clustering was performed using the hclust function in R, using log2 of gene expression values estimated by edgeR. Before clustering, the log2 expression values for each gene were centered by their median and rescaled so that the sum of the squares of the values equaled 1.0. Euclidean distance and the ward.D2 clustering method were used for the clustering of the samples and genes. The heatmap was plotted using the heatmap.2 function in R.

#### 4.5.3. Ingenuity Pathway Analysis (IPA) and the Integration of mRNA and miRNA Expression Data

Pathway enrichment analysis was performed using IPA [55] (QIAGEN Inc., https://www.qiagenbioinformatics.com/products/ingenuity-pathway-analysis (accessed on 1 December 2020)). The differentially expressed genes called by edgeR or differentially expressed genes and miRNAs identified from an integrated analysis of mRNA and miRNA expression were uploaded to IPA along with their log2 ratios to account for the direction of any changes in expression. IPA “Core Analysis” was performed using the “Ingenuity Knowledge Bases” to identify enriched pathways. All other parameters were set to their default values. To identify miRNAs whose upregulation or downregulation affected mRNA expression, we performed an integrated miRNA/mRNA gene expression analysis using the IPA “microRNA Target Filter” module. Pairs of miRNAs and affected genes were chosen if the targeting of a particular miRNA for a given mRNA had been experimentally validated or predicted with high confidence, and if the pair displayed inverse expression changes.

#### 4.5.4. Gene Expression Clustering and Gene Set Enrichment Analysis (GSEA)

The differentially expressed genes identified in the BK5.ATF3 mouse model were compared to those of human breast cancers in the TCGA database. Human homologs of mouse genes were identified, and their expression was compared to that of the differentially expressed genes (FDR < 0.05 and log2 fold change > 1 or < −1) identified in BK5.ATF3 mouse tumors. Normalized expression data for the human homologs were retrieved from the human breast cancer database (https://gdc.cancer.gov/node/877 (accessed on 1 December 2020)). A total of 547 tissue samples from breast cancer patients along with their PAM50 intrinsic breast cancer subtypes (Basal-like, Her2, LumA, LumB, and Normal) were included in the clustering analysis. The unsupervised hierarchical cluster was visualized as a heatmap and dendogram, which were plotted using the R programming package (heatmap.3), with the Spearman correlation and complete clustering settings.

Gene Set Enrichment Analysis (GSEA) was performed using GSEA software [56]. Expression values were estimated with the Bioconductor package edgeR using all genes as the expression dataset. The permutation type was set as “gene_set” and all the other parameters were set to their defaults. GSEA analysis identified enriched gene sets in the basal-like subtype of the breast cancer group. The human biological process gene sets in Gene Ontology were used for the comparison. The significantly enriched genes in the human gene sets (FDR < 0.05) were then intersected with their mouse homologs from the differentially expressed genes in BK5.ATF mice. The unsupervised hierarchical cluster heatmap was then plotted based on the list of the enriched genes as before.

#### 4.5.5. PAM50 Correlation Test

Differentially expressed human genes were retrieved from the TCGA BRCA RNA-Seq database. Each tumor type (Basal-like, Her2, LumA, LumB, and Normal-like) was compared to non-paired, adjacent normal samples with DESeq2 to generate a list of differentially expressed genes for each tumor type (FDR < 0.05). The change in expression of these human genes was correlated with the change in expression of the BK5.ATF3 mammary tumors, as described above for RNA-seq data analysis. Pearson’s correlation coefficient (r) and *p*-values were calculated using the log2FoldChange values obtained from the mouse differentially expressed genes, the human differentially expressed genes, and the PAM50 genes.

## 5. Conclusions

We characterized BK5.ATF3 mammary gland tumors and found these tumors have the molecular signature of human BLBC. In this BLBC mouse model, several EMT-related signaling pathways, including TGF-β, Wnt/β-catenin, Hedgehog, and Notch signaling pathways, are activated. These ATF3-induced tumors were also found to have reduced expression of *Mir145* and *Mir143*, leading to upregulation of the transcription factors *Klf4* and *Sox2* and the cancer stem cell-related gene *Kras*, respectively. Taken together, these results suggest that the BK5.ATF3 model can be used to further study molecular mechanisms of human BLBC to better understand its aggressiveness and its ability to metastasize to remote organ(s) via mammary stem cells. This model might also be used to identify additional molecular targets for more effective therapeutic treatments for this deadly cancer.

## Data Availability

The datasets generated and analyzed for array and RNA-Seq in this publication have been deposited in NCBI’s Gene Expression Omnibus (GEO) [57] and are accessible through GEO Series accession numbers GSE162793 (https://www.ncbi.nlm.nih.gov/geo/query/acc.cgi?acc=GSE162793 (accessed on 1 December 2020)) and GSE162804 https://www.ncbi.nlm.nih.gov/geo/query/acc.cgi?acc=GSE162804 (accessed on 1 December 2020)).

## Supplementary Materials

The following are available online at https://www.mdpi.com/1422-0067/22/5/2353/s1.

## Abbreviations

ATF3: activating transcription factor 3; BLBC: basal-like breast cancer; RNA-Seq: Ribonucleic acid sequencing; miRNA: microRNA; BK5: bovine cytokeratin-5; RT-qPCR: reverse transcriptase-quantitative polymerase chain reaction; FDR: false discovery rate; FC: fold change; IPA: Ingenuity Pathway Analysis; hclust: hierarchical clustering; GSEA: Gene Set Enrichment Analysis; PAM50: Prediction Analysis of Microarray 50; TCGA: The Cancer Genome Atlas Network; DE and DEGs: differentially expressed genes; EMT: epithelial mesenchymal transition; GO: Gene Ontology; TNBC: triple-negative breast cancer; MSigDB: Molecular Signatures Database; GEO: Gene Expression Omnibus.
