# Supplementary material for: ATF3-Induced Mammary Tumors Exhibit Molecular Features of Human Basal-Like Breast Cancer
Source: Int J Mol Sci. 2021 Feb 26;22(5):2353. doi: 10.3390/ijms22052353 (PMC7956570; doi:10.3390/ijms22052353)
Supplement: Supplementary file 1 [file ijms-22-02353-s001.zip › ATF3 Supplementary_IJMS Submission/Supplementary Table 1 .docx]

**Supplementary Table 1.** Differentially expressed miRNA genes

| **Gene Name**^a^ | **Differential Expression** | | |
| --- | --- | --- | --- |
|  | Fold Change by Microarray^b^ | Adjusted P-value^c^ | qPCR Validation^d^ |
| *Mir31* | 16.54 | 2.44 X 10^-15^ | 72.0 |
| *Mir203* | 8.95 | 3.44 X 10^-17^ |  |
| *Mir205* | 7.49 | 4.31 X 10^-15^ | 7.65 |
| *Mir31** | 6.66 | 7.07 X 10^-07^ |  |
| MIR34b-5p | 3.97 | 1.07 X 10^-06^ | 5.39 |
| *Mir96* | 3.89 | 4.81 X 10^-07^ | 2.90 |
| *Mir183* | 3.81 | 1.80 X 10^-09^ | 5.63 |
| *Mir138* | 3.51 | 4.27 X 10^-06^ |  |
| *Mir21* | 3.45 | 5.50 X 10^-12^ | 3.29 |
| *Mir503* | 3.44 | 2.33 X 10^-08^ |  |
| *Mir18a* | 3.22 | 2.44 X 10^-07^ | 3.77 |
| *Mir34a* | 3.18 | 6.31 X 10^-10^ | 4.99 |
| *Mir182* | 2.79 | 1.53 X 10^-05^ |  |
| *Mir223* | 2.42 | 6.90 X 10^-07^ |  |
| *Mir26b* | 0.40 | 8.25 X 10^-07^ |  |
| *Mir29b* | 0.37 | 1.34 X 10^-07^ |  |
| *Mir30a* | 0.36 | 1.12 X 10^-06^ |  |
| *Mir26a* | 0.36 | 9.24 X 10^-06^ |  |
| *Mir30e* | 0.35 | 1.30 X 10^-08^ |  |
| MIR126-3p | 0.34 | 1.72 X 10^-08^ |  |
| *Mir30c* | 0.32 | 1.87 X 10^-11^ | 0.26 |
| *Mir143* | 0.31 | 7.81 X 10^-12^ | 0.19 |
| *Mir30b* | 0.31 | 1.32 X 10^-11^ | 0.05 |
| Mir126-5p | 0.30 | 1.54 X 10^-07^ |  |
| *Mir145* | 0.29 | 8.34 X 10^-09^ | 0.17 |
| *Mir29c* | 0.25 | 4.28 X 10^-10^ |  |
| *Mir365* | 0.25 | 2.08 X 10^-07^ |  |
| *Mir144* | 0.25 | 1.80 X 10^-09^ |  |
| *Mir101a* | 0.24 | 8.34 X 10^-09^ |  |
| *Mir150* | 0.22 | 2.31 X 10^-08^ |  |
| *Mir378* | 0.22 | 3.49 X 10^-09^ | 0.16 |
| *Mir193* | 0.22 | 8.85 X 10^-11^ |  |
| *Mir451* | 0.18 | 8.85 X 10^-11^ | 0.17 |
| *Mir708* | 0.15 | 1.19 X 10^-11^ |  |

^a^microRNA gene names in mouse are based on nomenclature guidelines established by Desvignes et al. (Desvignes, Batzel et al. 2015).

^b^Fold changes are in log2ratio of miRNA between mammary tumor tissues over matched adjacent normal mammary glands.

^c^miRNAs were considered significantly differentially expressed if the adjusted p-value was <5X10-4.

^d^Fold changes by qPCR validation are also in log2ratio of tumor over adjacent normal.
