# Supplementary figures and images for: ATF3-Induced Mammary Tumors Exhibit Molecular Features of Human Basal-Like Breast Cancer
Source: Int J Mol Sci. 2021 Feb 26;22(5):2353. doi: 10.3390/ijms22052353 (PMC7956570; doi:10.3390/ijms22052353)

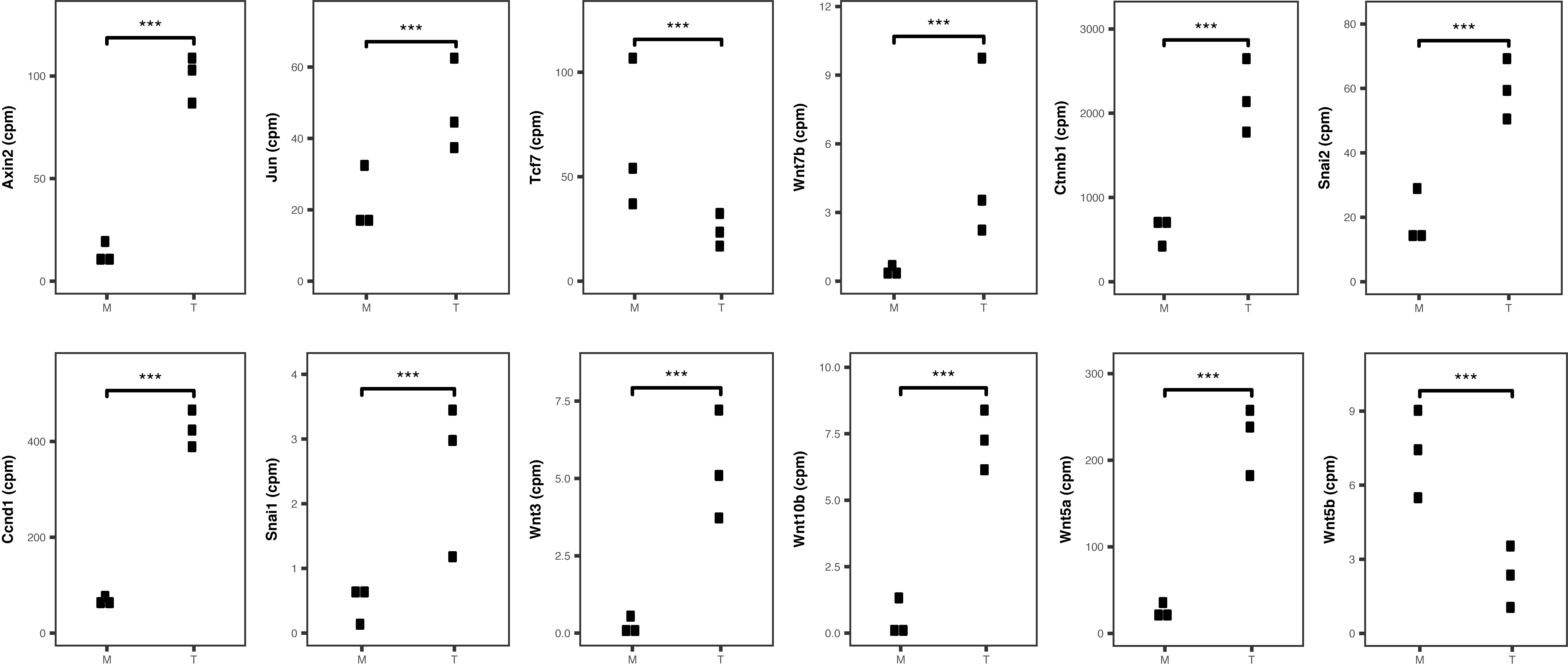

Supplement: Supplementary file 1 [file ijms-22-02353-s001.zip › ATF3 Supplementary_IJMS Submission/Sup_Fig1.tif]

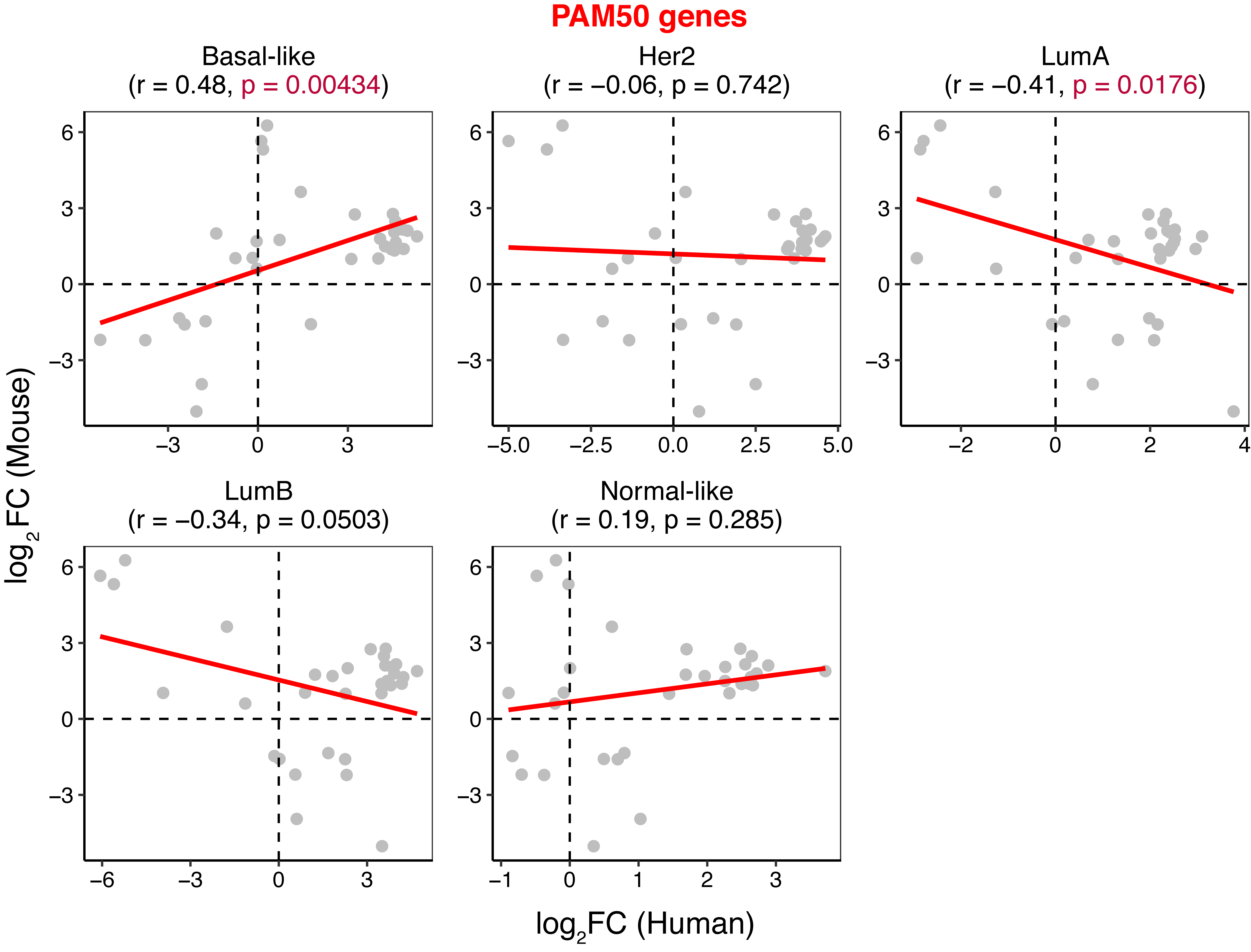

Supplement: Supplementary file 1 [file ijms-22-02353-s001.zip › ATF3 Supplementary_IJMS Submission/Sup_Fig2.tif]

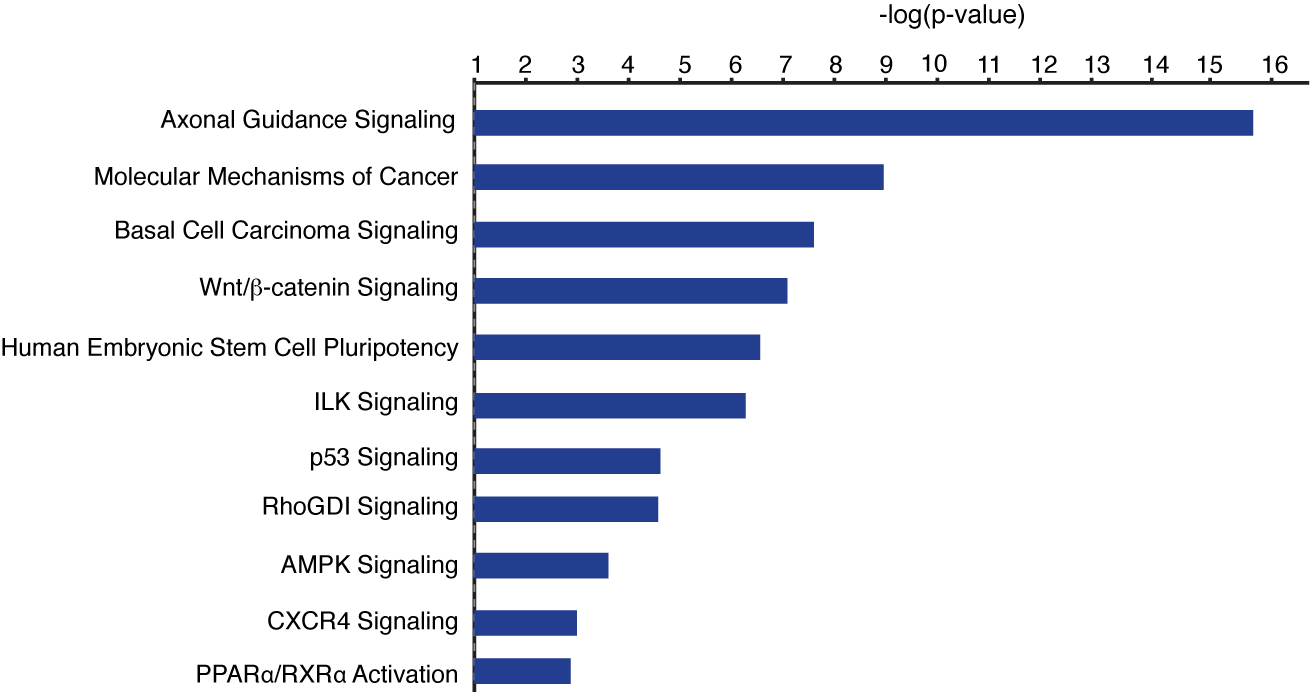

Supplement: Supplementary file 1 [file ijms-22-02353-s001.zip › ATF3 Supplementary_IJMS Submission/Sup_Fig3.tif]

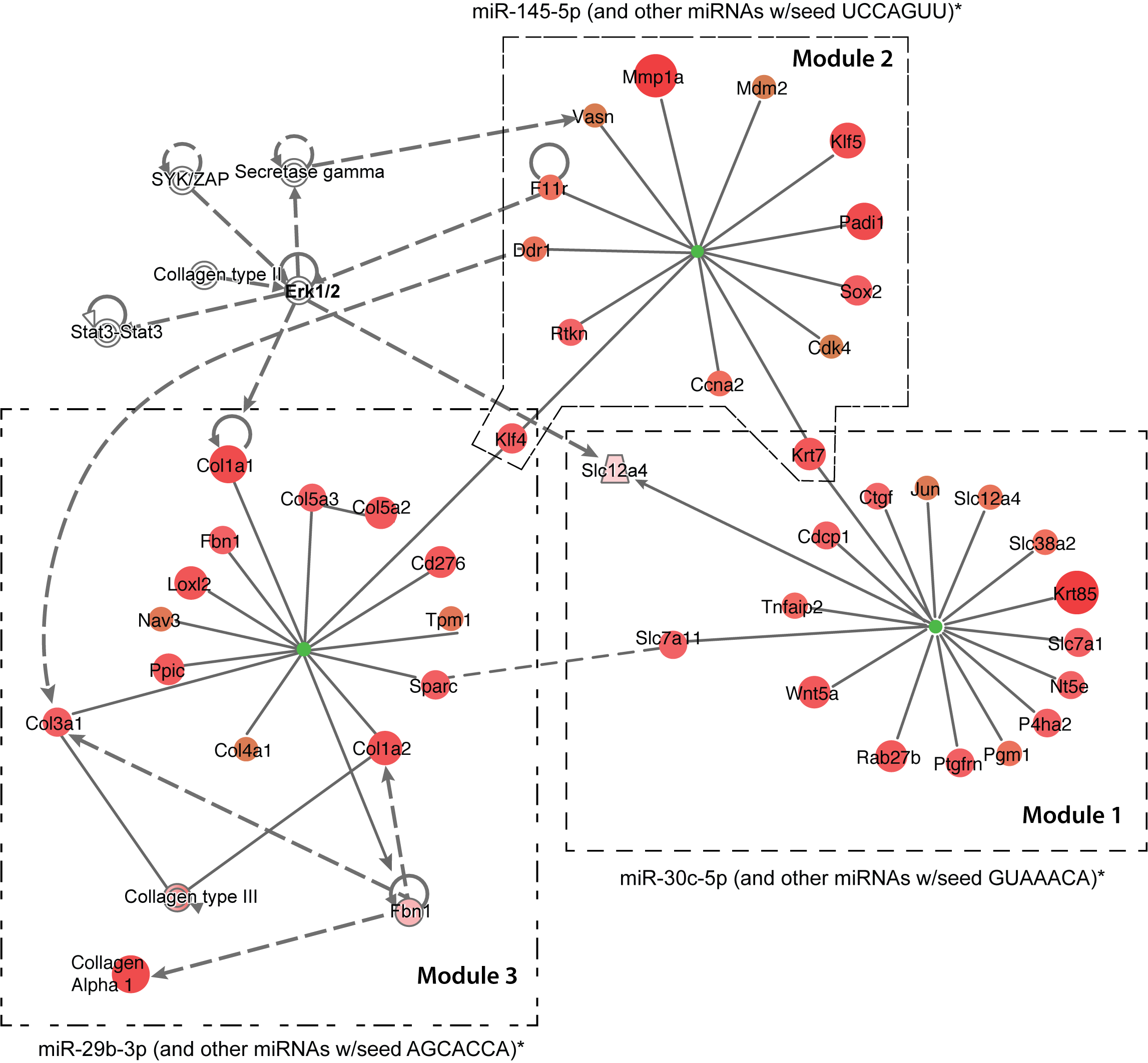

Supplement: Supplementary file 1 [file ijms-22-02353-s001.zip › ATF3 Supplementary_IJMS Submission/Sup_Fig4.tif]

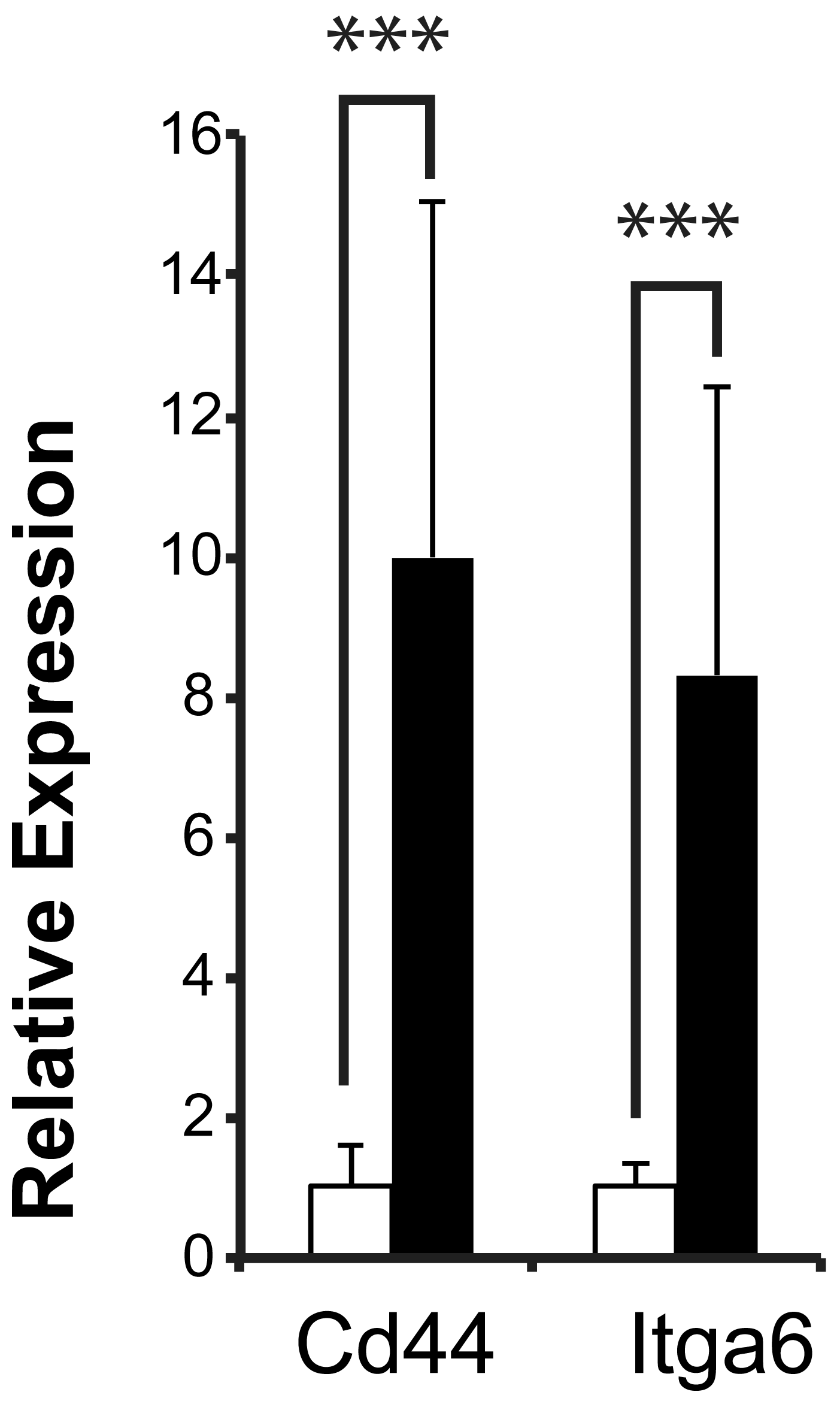

Supplement: Supplementary file 1 [file ijms-22-02353-s001.zip › ATF3 Supplementary_IJMS Submission/Sup_Fig5.tif]
